# Gene‐Based Clustering Identifies QSOX1 and IL1RAP as Biomarkers of Metabolic Dysfunction‐Associated Steatotic Liver Disease

**DOI:** 10.1111/liv.70823

**Published:** 2026-07-29

**Authors:** Wenfeng Ma, Jinrong Huang, Benqiang Cai, Mumin Shao, Xuewen Yu, Mikkel Breinholt Kjær, Minling Lv, Xin Zhong, Shaomin Xu, Bolin Zhan, Qun Li, Qi Huang, Mengqing Ma, Lei Cheng, Yonglun Luo, Henning Grønbæk, Xiaozhou Zhou, Lin Lin

**Affiliations:** ^1^ Department of Liver Disease Shenzhen Traditional Chinese Medicine Hospital Shenzhen Guangdong China; ^2^ Department of Biomedicine Aarhus University Aarhus Denmark; ^3^ Steno Diabetes Center Aarhus Aarhus University Hospital Aarhus Denmark; ^4^ Department of Liver Disease The Fourth Clinical Medical College of Guangzhou University of Chinese Medicine Shenzhen China; ^5^ Department of Pathology Shenzhen Traditional Chinese Medicine Hospital Shenzhen Guangdong China; ^6^ Department of Pathology The Fourth Clinical Medical College of Guangzhou University of Chinese Medicine Shenzhen China; ^7^ Department of Clinical Medicine Aarhus University Aarhus Denmark; ^8^ Department of Hepatology and Gastroenterology Aarhus University Hospital Aarhus Denmark

**Keywords:** interleukin‐1 receptor accessory protein, metabolic dysfunction‐associated steatotic liver disease, non‐invasive biomarker, quiescin sulfhydryl oxidase 1, RNA sequencing data integration

## Abstract

**Background and Aims:**

Metabolic dysfunction‐associated steatotic liver disease (MASLD) is a progressive liver disease that ranges from simple steatosis to inflammation, fibrosis and cirrhosis. To address the unmet need for new MASLD biomarkers, we aimed to identify candidate biomarkers using publicly available RNA sequencing (RNA‐seq) and proteomics data.

**Methods:**

An approach involving unsupervised gene clustering was performed using homogeneously processed and integrated RNA‐seq data of 625 liver specimens to screen for MASLD biomarkers, in combination with public proteomics data from healthy controls and MASLD patients. Additionally, we validated the results in the MASLD and healthy cohorts using enzyme‐linked immunosorbent assay (ELISA) of plasma and immunohistochemical staining (IHC) of liver samples.

**Results:**

We generated a database (https://dreamapp.biomed.au.dk/NAFLD/) for exploring gene expression changes along MASLD progression to facilitate the identification of genes and pathways involved in the disease's progression. Through cross‐analysis of the gene and protein clusters, we identified 38 genes as potential biomarkers for MASLD severity. Up‐regulation of Quiescin sulfhydryl oxidase 1 (*QSOX1*) and down‐regulation of Interleukin‐1 receptor accessory protein (*IL1RAP*) were associated with increasing MASLD severity in RNA‐seq and proteomics data. Particularly, the QSOX1/IL1RAP ratio in plasma demonstrated effectiveness in diagnosing MASLD, with an area under the receiver operating characteristic (AUROC) of up to 0.95 as quantified by proteomics profiling and an AUROC of 0.82 with ELISA.

**Conclusions:**

We discovered a significant association between the levels of QSOX1 and IL1RAP and MASLD severity. Furthermore, the QSOX1/IL1RAP ratio shows promise as a non‐invasive biomarker for diagnosing MASLD and assessing its severity.

## Highlights

- RNA‐seq data from 625 liver specimens comprising healthy controls and patients with MASLD with increasing severity were utilized for screening of MASLD biomarkers.
- An unsupervised method for clustering genes based on the similarity of gene expression trajectory across all samples enhanced the discovery of novel effective non‐invasive MASLD biomarkers.
- QSOX1, IL1RAP and especially the QSOX1/IL1RAP ratio were found to be associated with MASLD severity.
- The high sensitivity of the QSOX1/IL1RAP ratio in predicting MASLD severity was validated with plasma proteomics quantification (AUROC = 0.95) and ELISA (AUROC = 0.82) in two independent patient cohorts.

## Lay Summary

This study aimed to find non‐invasive biomarkers for metabolic dysfunction‐associated steatotic liver disease (MASLD). Researchers utilized a new gene clustering method to analyse RNA‐seq data from 625 liver samples. The identified biomarkers were further validated using plasma proteomics profiling, enzyme‐linked immunosorbent assay (ELISA) and liver immunohistochemical staining (IHC) in three separate groups of healthy controls and MASLD patients. The study revealed that the levels of QSOX1 were elevated while IL1RAP levels were reduced with increasing severity of MASLD. Importantly, the ratio of QSOX1 to IL1RAP expression in plasma showed promise as a non‐invasive diagnostic tool for assessing the severity of MASLD, eliminating the reliance on liver biopsy.

**Abbreviations**
AUROC: area under the receiver operating characteristic
avg_log2FC: log fold‐change of the average expression between the two groups
BMI: body mass index
CK18: circulating keratin 18 fragments
ECM: extracellular matrix
ELISA: enzyme‐linked immunosorbent assay
F: fibrosis score
FIB‐4: Fibrosis‐4
GEO: Gene Expression Omnibus
GO: Gene Ontology
GRCh37: Genome Reference Consortium Human Build 37
HCC: hepatocellular carcinoma
IHC: immunohistochemistry staining
IL1RAP: Interleukin‐1 receptor accessory protein
lg: log10
MASLD: Metabolic dysfunction‐Associated Steatotic Liver Disease
MASLD‐DB: MASLD gene expression database
MASLD_ngt: MASLD with normal glucose tolerance
MASLD_T2D: MASLD with type 2 diabetes
N: NAS score
NAS: NAFLD activity scores
NASH: non‐alcoholic Steatohepatitis
ncRNAs: non‐coding RNAs
PCA: Principal components analysis
QSOX1: Quiescin sulfhydryl oxidase 1
RNA‐seq: RNA sequencing
scRNA‐seq: single‐cell RNA sequencing
snRNA‐seq: single‐nuclei RNA sequencing
SZTCMH: Shenzhen Traditional Chinese Medicine Hospital, China
THBS2: thrombospondin 2
TMM: TRIMMED MEAN of M‐values
TPM: transcript per million

## 1 Introduction

Metabolic dysfunction‐Associated Steatotic Liver Disease (MASLD) is recognized as the hepatic manifestation of the metabolic syndrome, with an estimated global prevalence of around 25%–32% [1, 2]. The severity of this liver disease ranges from simple steatosis to Metabolic dysfunction‐Associated Steatohepatitis (MASH) with inflammation and fibrosis, which can progress to MASH‐induced cirrhosis and increase the risk of hepatocellular carcinoma (HCC).

Liver biopsy is currently the gold standard for histological diagnosis of MASLD despite its associated side effects such as pain, bleeding and rare mortality. To address these drawbacks and reduce costs, there is still an unmet need for novel, precise and cost‐effective imaging tools and non‐invasive biomarkers [3]. Moreover, non‐invasive biomarkers are highly needed for replacing repeated liver biopsies when assessing liver histology during pharmacological interventions. Existing MASLD biomarkers primarily focus on steatosis (e.g., SteatoTest or the lipid accumulation product), inflammation (e.g., circulating keratin 18 fragments [CK18], soluble CD163) or fibrosis (e.g., ELF, FibroTest or Pro‐C3 tests) [4, 5, 6, 7, 8]. Despite advancements in biomarker technology, development and evaluation, an ideal biomarker for the diagnosis, prognosis and assessment of treatment effects in MASLD has yet to be identified.

The traditional RNA‐seq analysis approach, which relies on established tools such as edgeR [9], DESeq2 [10] and Cufflinks [11], primarily focuses on identifying differentially expressed genes (DEGs) through pairwise comparisons [12]. However, for conditions like MASLD, which involve a complex scoring system and a continuous range of histological variations, this approach has its limitations. MASLD doesn't involve transitioning between distinct states but represents a dynamic progression through constant histopathological changes. Pairwise comparisons oversimplify the intricate genetic alterations that occur throughout MASLD's development. What's required is a more advanced analytical method capable of capturing the gradual and overlapping gene expression changes across the entire spectrum of MASLD. Such an approach would offer a comprehensive representation of MASLD's complexity and enhance our understanding of its progression. In recent years, advancements in technologies such as RNA sequencing (RNA‐seq), single‐cell analysis and spatial transcriptomics have provided deeper insights into the molecular and cellular processes involved in MASLD progression [13, 14, 15]. Large‐scale profiling efforts, combined with targeted validation approaches, have led to the discovery of potential biomarkers [16, 17]. However, the majority of available RNA‐seq data are derived from smaller cohorts of MASLD patients, which limits the comprehensive understanding of MASLD severity.

In this study, modularity optimization methods were utilized to cluster genes by employing a graph‐based strategy, taking into account the gene expression patterns throughout the progression of MASLD. We propose that integrating and analysing these datasets with the unbiased gene‐based profiling strategy will provide further insights into the molecular progression of MASLD and the identification of biomarkers associated with MASLD severity. In the present study, we identified over 300 MASLD biomarkers by integrating and analysing RNA‐seq data from 625 liver samples, including their NAFLD activity scores (NAS) and fibrosis scores, along with public proteomics data. We further validated these findings in two independent MASLD cohorts, demonstrating the potential of the QSOX1/IL1RAP ratio as a non‐invasive biomarker for diagnosing MASLD and assessing its severity.

## 2 Materials and Methods

### 2.1 Data Collection

Genome‐wide RNA‐seq data of human MASLD and associated healthy controls were collected from the NCBI GEO (https://www.ncbi.nlm.nih.gov/gds, access date until May 2022). Only datasets that provided detailed NAS and fibrosis scores were included for further investigation, including seven datasets (GSE105127 [18], GSE107650 [19], GSE126848 [20], GSE130970 [21], GSE135251 [22, 23], GSE162694 [24] and GSE167523 [25] (Table S1)).

### 2.2 Data Normalization

**FIGURE 1:**
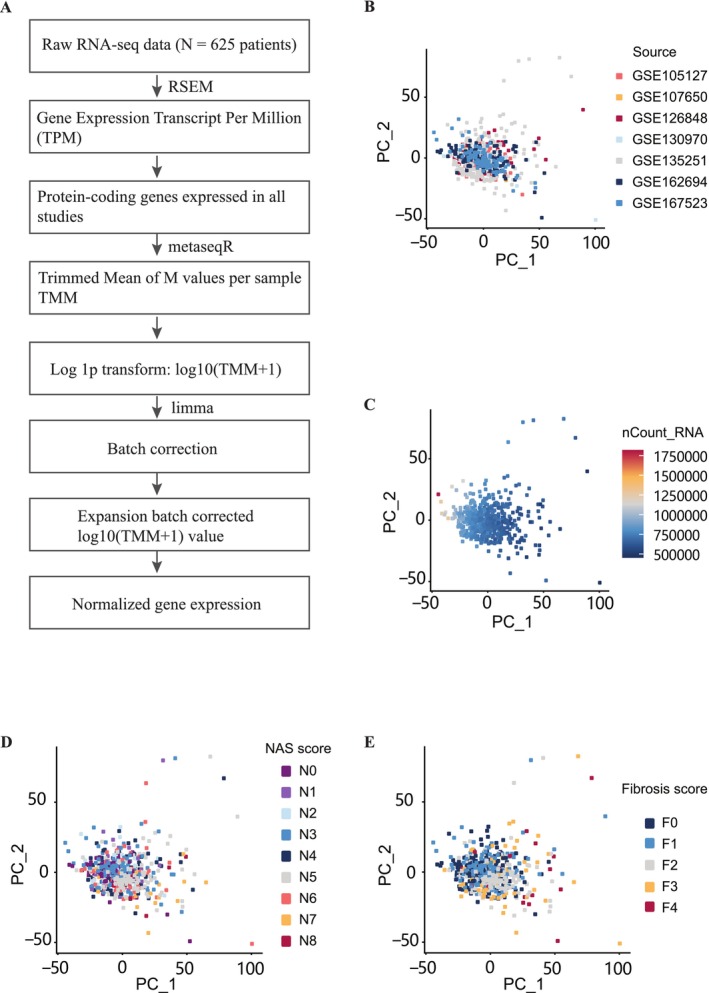
RNA sequencing data processing, integration and analysis. (A) Illustration of data processing. (B) Principal component analysis (PCA) based on the origin of datasets. (C) PCA based on normalized RNA abundance (nCount). (D) PCA based on NAS score. (E) PCA based on fibrosis scores.

The SRA‐formatted data were converted into FASTQ format using ‘SraToolkit’ (sratoolkit.2.8.2‐1‐centos_linux64) (https://github.com/ncbi/sra‐tools). Sequencing reads were aligned to the hg19 UCSC RNA sequences Genome Reference Consortium Human Build 37 (GRCh37) using ‘bowtie2’ (bowtie2‐2.2.5) (https://rnnh.github.io/bioinfo‐notebook/docs/bowtie2.html). Only protein‐coding transcripts were considered, and Transcript Per Million (TPM) values were obtained by transforming the mapped transcript reads using ‘RSEM’ (rsem‐1.2.12) (https://github.com/deweylab/RSEM). Then, TPM values were then subjected to Trimmed Mean of M‐values (TMM) normalization across all samples using ‘metaseqR’ (metaseqR 1.12.2) [26]. The data from various sources involved in this study were integrated and log1p‐transformed, followed by batch correction using the ‘removeBatchEffect’ function in the R package ‘limma’ (limma 3.54.2) (https://kasperdanielhansen.github.io/genbioconductor/html/limma.html) [27]. Subsequently, the data were expanded (10^x) for further analysis (Figure 1A).

### 2.3 RNA‐Seq Data Analysis and Database Construction

After normalization and batch correction, the RNA‐seq data were subjected to Principal Components Analysis (PCA) and unsupervised clustering using the R package ‘Seurat’ (Seurat‐4.3.0) (https://satijalab.org/seurat/articles/get_started.html). We utilized the “LogNormalize” method for global‐scaling normalization, which normalized the feature expression measurements across different samples for each gene by the total expression. The normalized values were multiplied by a scale factor (default: 10000) and log1p‐transformed. Subsequently, scaling was applied to the identified variable features (default: 2000). PCA was then performed on the scaled data, with a default setting of computing and storing 50 Principal Components (PCs). To cluster the genes, we employed modularity optimization techniques using a graph‐based clustering approach. The dimensions of reduction were set to 1:20, and the resolution parameter was set to 2.3 [28].

To show the gene expression variation during the development of MASLD associated with both NAS and fibrosis scores, we generated an RNA‐seq database using ‘ShinyCell’ (https://github.com/SGDDNB/ShinyCell). This database was deployed at https://dreamapp.biomed.au.dk/NAFLD/.

### 2.4 Proteomics Data Collection and Analysis

The proteomics cohort dataset (PXD011839) includes 10 healthy controls, 10 MASLD patients with normal glucose tolerance (MASLD_ngt), 10 MASLD patients with type 2 diabetes (MASLD_T2D) and 10 MASLD patients with cirrhosis [29]. We performed the statistical analysis using R‐4.3.0 on the dataset (PMID: 30824564, EV1, table 4) (https://pmc.ncbi.nlm.nih.gov/articles/instance/6396370/bin/MSB‐15‐e8793‐s003.xlsx). The MASLD_ngt and MASLD_T2D groups were merged into a single MASLD group, resulting in three groups: healthy controls, MASLD and cirrhosis.

Similar to RNA‐seq analysis, we used ‘Seurat’ (Seurat‐4.3.0) and employed the ‘LogNormalize’ method for global‐scaling normalization. This method normalized the feature expression measurements across different samples for each protein by the total expression. The normalized values were multiplied by a scale factor (default: 10000) and log1p‐transformed. Subsequently, scaling was applied to the identified variable features (default: 2000). PCA was performed on the scaled data, with a total of 39 Principal Components (PCs) computed and stored. Additionally, we calculated the log fold‐change of the average expression between two groups (avg_log2FC) by comparing the health and MASLD groups, as well as the MASLD and cirrhosis groups. By setting avg_log2FC > 0, we selected up‐ and down‐regulated proteins associated with increasing severity of MASLD.

### 2.5 Validation of QSOX1 and IL1RAP as Biomarkers in MASLD

Our objective was to investigate whether *QSOX1* and *IL1RAP* gene expression levels, as well as their encoded proteins, could predict the histological severity of MASLD. To address this question, we examined the plasma concentrations of QSOX1 and IL1RAP in the proteomics data of healthy and MASLD cohorts. Additionally, we conducted enzyme‐linked immunosorbent assay (ELISA) tests for plasma QSOX1/IL1RAP in a cohort comprising healthy subjects and MASLD patients recruited at Shenzhen Traditional Chinese Medicine Hospital, China (SZTCMH).

#### 2.5.1 Human Samples

A total of 28 ultrasound‐proven adult MASLD patients, including MASLD_ngt and MASLD_T2D, and 14 healthy controls were enrolled from SZTCMH. Other diagnoses and etiologies, such as excessive alcohol consumption, viral hepatitis, autoimmune liver disease and the use of steatogenic compounds, were excluded. Archived plasma samples were collected between October and December 2022. Informed consent was obtained from the healthy subjects and patients with MASLD, following the approved clinical protocols of the Ethical Committee of SZTCMH. Clinical information, including body mass index (BMI) and standard biochemistry (liver, kidney, haematology) with metabolic profiling (glucose, insulin, lipids), was collected. Fibroscan with controlled attenuation parameter (CAP) values was performed to assess fibrosis and steatosis. Clinical and biochemical information for the healthy controls and patients with MASLD can be found in Tables S5 and S6.

For immunohistochemical staining (IHC), 12 fixed liver tissues were collected from archived histological samples at SZTCMH between 2014 and 2023. These samples were scored based on the NAS score (N0 to N8) and fibrosis score (F0 to F4) by two pathologists (MMS and XWY). Six samples were from patients with mild MASLD (N0–4, F0–2), and six were from patients with severe MASLD (N5–8, F3–4). The clinical study was approved by the Ethical Committee of SZTCMH, and the approved clinical protocols adhere to the Helsinki Declaration (No. K2022‐174‐01).

#### 2.5.2 ELISA

Blind ELISA tests were conducted on the collected plasma samples. Randomly assigned sample identifiers and positions were used to ensure blindness to the clinical information and MASLD stages. The levels of QSOX1 and IL1RAP were measured using QSOX1 ELISA Kits (Catalogue No. YJ145587, Lot No. 12/2022 from Enzyme‐linked Biotechnology, Shanghai, China) and IL1RAP ELISA Kits (Catalogue No. YJ130558, Lot No. 12/2022 from Enzyme‐linked Biotechnology, Shanghai, China), respectively. The measurements followed the manufacturer's instructions and the absorbance values were measured at 450 nm. To ensure the reliability of the ELISA Kits, a pre‐experiment was conducted three times before the formal experiment.

#### 2.5.3 Immunohistochemistry Assay

We examined the association of QSOX1 and IL1RAP with human MASLD severity by performing IHC on formalin‐fixed and paraffin‐embedded liver sections from six patients with mild MASLD (N0‐4, F0‐2), and six patients with severe MASLD (N5‐8, F3‐4). The 3 μm‐thick paraffin sections were deparaffinized and rehydrated with distilled water. Antigen retrieval was carried out using pH 9.0 EDTA buffer, followed by 20 min of boiling and washing with 1× PBS. Subsequently, the slides were blocked with 1% bovine serum albumin in 1× PBS for 15 min and then incubated overnight at 4°C with QSOX1 (Rabbit anti‐human, Catalogue No. Ab235444, Lot No. GR3386311‐2 from Abcam) or IL1RAP (Rabbit anti‐human, Catalogue No. 35605, Lot No. 4926 from Sabbiotech) antibodies at a concentration of 20 μg/mL. The following day, the slides were washed with 1× PBS and incubated with Goat anti‐rabbit IgG H&L (Catalogue No. Ab205718, Lot No. ab205718 from Abcam) for 15 min at room temperature, followed by another wash with 1× PBS. The images were captured using a light microscope and 3DHISTECH digital scanner (https://www.3dhistech.com/).

The IHC results were analysed using the software tool ‘ImagineJ (Fiji)’. To prevent potential bias, we randomly selected five locations of the same size from each sample at 20× magnification using 3DHISTECH CaseViewer_2.4 (https://www.3dhistech.com/solutions/caseviewer/). Using ‘ImagineJ’, we applied the ‘Colour Deconvolution’ tool with vectors = [H DAB]; followed by selecting the Colour_2 pictures and running ‘8‐bit’. Standard thresholds were used (QSOX1: setThreshold (60, 230), IL1RAP: setThreshold (94, 214)) [30, 31]. The average integrated density from the five sites was calculated and used as the integrated density value for each sample, which was then subjected to statistical analysis.

### 2.6 Statistical Analysis

The significance for all statistical tests was two‐sided, with *p* < 0.05. All data analysis was presented in the plots using R‐4.3.0, and MedCalc was used to calculate the AUROC, sensitivity, specificity, optimal cutoff value and sample size.

## 3 Results

### 3.1 Overview of RNA‐Seq Data and MASLD Patient Cohorts

After applying stringent filtering criteria based on the availability of histological NAS and fibrosis scores, five datasets including GSE115193 [32], GSE134422 [33], GSE135448 [34], GSE160016 [35] and GSE164441 [36] were excluded from the analysis, while seven datasets (GSE105127 [18], GSE107650 [19], GSE126848 [20], GSE130970 [21], GSE135251 [22, 23], GSE162694 [24] and GSE167523 [25]) were included. These datasets collectively comprise 81 healthy controls (including healthy obese individuals) (N0F0) and 544 patients with MASLD. The severity of MASLD was classified based on the NAS score (ranging from N1 to N8) and the fibrosis score (ranging from F0 to F4) using the scoring systems proposed by Brunt [37] and Kleiner [38], respectively (Table 1, Table S1). A positive correlation was observed between NAS and fibrosis scores (Pearson *R* = 0.64, *p* = 4.94E‐74, Table 1), indicating an association with MASLD severity.

**TABLE 1:** Characteristics of the populations studied: A total of 625 human liver samples of the full histological range from normal, MASLD, MASH to cirrhosis with NAS (N) and fibrosis (F) scores provided in the database or the original articles.

Sample distribution of NAS‐ and F‐scores	F0	F1	F2	F3	F4	Age (years)	BMI (kg/m^2^)
N0 (*n*)	81	1	1	—	1	46.4 ± 10.0	35.4 ± 9.1
N1 (*n*)	29	9	1	—	1	42.8 ± 12.7	36.1 ± 6.6
N2 (*n*)	27	10	1	—	1	51.9 ± 5.8	35.1 ± 6.0
N3 (*n*)	46	72	9	5	3	49.8 ± 9.4	34.6 ± 8.9
N4 (*n*)	30	19	20	15	3	49.6 ± 9.5	33.9 ± 4.6
N5 (*n*)	7	41	75	20	5	52.4 ± 11.7	32.5 ± 5.5
N6 (*n*)	1	18	17	18	3	53.2 ± 8.5	34.5 ± 5.6
N7 (*n*)	—	1	14	11	1	49.4 ± 9.8	36.3 ± 7.2
N8 (*n*)	—	—	2	4	2	54.0 ± 0.0	31.3 ± 0.0
Age (years)	46.6 ± 9.6	49.0 ± 10.6	53.2 ± 11.3	54.7 ± 6.0	54.8 ± 5.4		
BMI (kg/m^2^)	37.1 ± 8.3	32.4 ± 5.6	33.1 ± 7.0	32.5 ± 2.9	32.6 ± 2.2		
Gender (*n*, male/female)	221 (105/116)^a^	171 (108/63)^a^	140 (64/76)^a^	73 (35/38)^a^	20 (11/9)^a^		

^a^ The ratio of gender was estimated according to the gender ratio in the original articles. BMI: body mass index (data are mean ± SD).

### 3.2 Normalization and Integration of RNA‐Seq Data

To address the issue of batch effects resulting from differences in sequencing technology and studies, we processed the integrated data as depicted in Figure 1A. Genes with low expression were filtered out, resulting in a total of 17 946 protein‐coding genes. Principal component analysis (PCA) demonstrated that normalization effectively eliminated noticeable batch effects (Figure 1B). Moreover, neither the NAS nor fibrosis scores appeared to be the main factors contributing to sample separation (Figure 1D,E). Instead, the normalized RNA abundance (nCount) in each sample emerged as the key component influencing transcriptome profiles (Figure 1C).

### 3.3 Unsupervised Gene Clustering Identifies Clusters of Genes Associated With MASLD Severity

**FIGURE 2:**
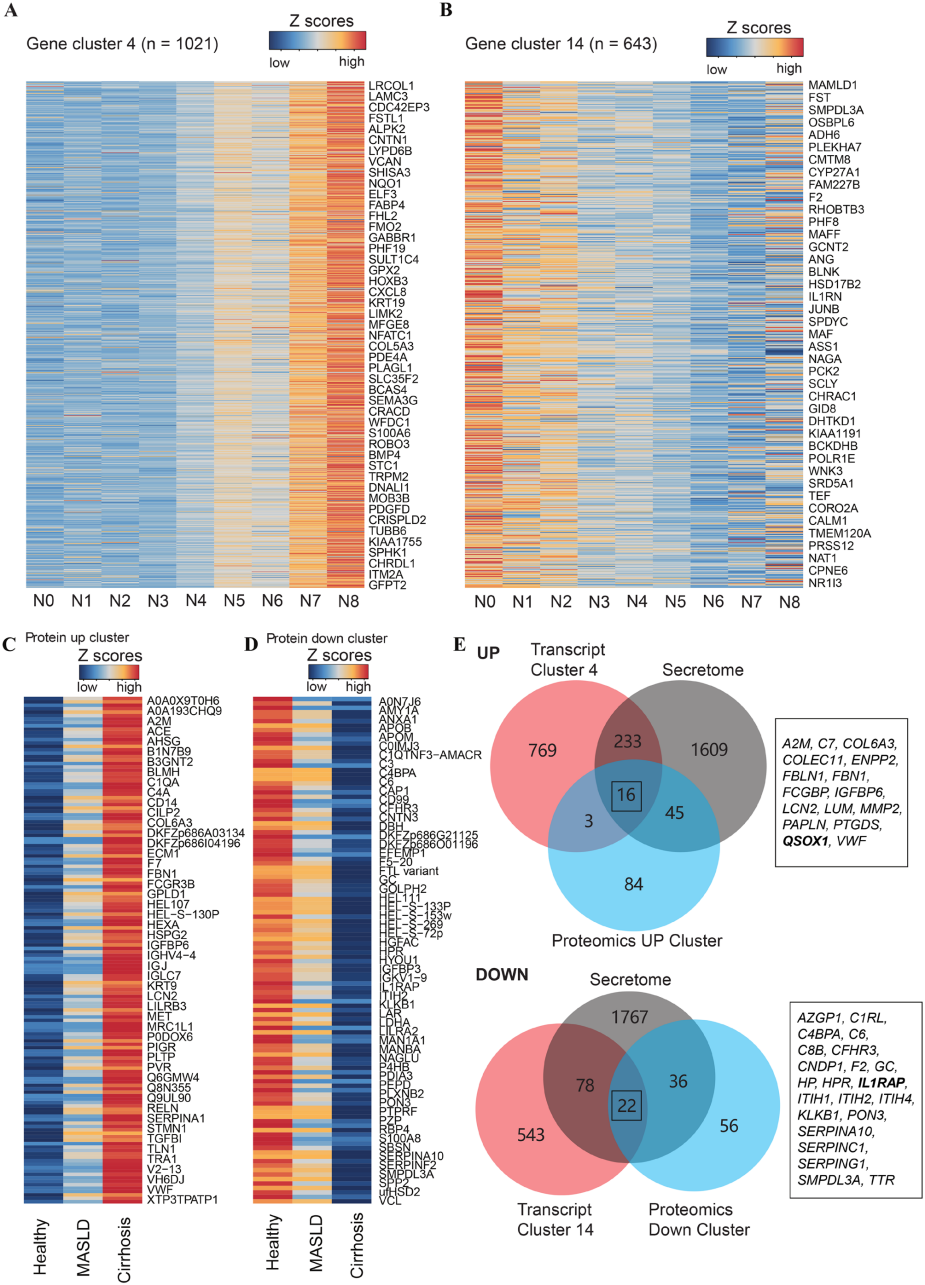
Integrative transcriptome and proteomics analysis to identify MASLD biomarkers. (A) Heatmap presentation of 1021 up‐regulated genes in cluster 4 associated with increasing NAS scores. (B) Heatmap presentation of 643 down‐regulated genes in cluster 14 associated with increasing NAS scores. (C) Protein cluster of 148 up‐regulated proteins associated with increasing MASLD severity in PXD011839. (D) Protein cluster of 114 down‐regulated proteins associated with increasing MASLD severity in PXD011839. (E) (UP) Venn diagram showing 16 overlapping genes between up‐regulated genes identified by RNA‐seq, up‐regulated proteins in the plasma, and secreting proteins. (DOWN) Venn diagram showing 22 overlapping genes between down‐regulated genes identified by RNA‐seq, down‐regulated proteins in the plasma, and secreting proteins.

To identify genes associated with MASLD severity, we utilized a previously developed unsupervised gene clustering method based on the similarity of gene expression patterns across each sample [39]. We employed gene clustering, grouping genes according to their expression patterns during the progression of MASLD. By setting a resolution of 2.3, we identified a total of 37 gene clusters (Figure S1). Notably, cluster 4, consisting of 1021 genes, consistently exhibited increased expression with higher NAS and fibrosis scores (Figure 2A, Figure S2A). Conversely, cluster 14, comprising 643 genes, showed decreased expression with increasing NAS and fibrosis scores (Figure 2B, Figure S2B). As illustrated in figures (Figure 2A,B, Figure S2A–D), this approach efficiently clustered genes into distinct groups based on their expression patterns across MASLD severity stages. It offers a more structured depiction of gene expression variations, enabling a deeper understanding of MASLD's molecular pathogenesis. Through visualizing and categorizing these gene expression changes, we can acquire a more comprehensive insight into the underlying mechanisms and factors that propel MASLD progression.

To explore the biological functions of these gene clusters, we performed Gene Ontology (GO) analysis using the R package ‘ClusterProfiler’ (ClusterProfiler‐4.8.0). Specifically, we focused on cluster 4, which consisted of up‐regulated genes. The GO analysis revealed significant enrichment of genes involved in the fibrosis‐related process, such as extracellular matrix (ECM) organization (*p*.adjust = 9.77E‐34), extracellular structure organization (*p*.adjust = 9.77E‐34), external encapsulating structure organization (*p*.adjust = 1.09E‐33) and cell‐substrate adhesion (*p*.adjust = 3.35E‐17). Notably, the expression of multiple genes involved in the ECM processes, such as *COL5A3*, *FBLN5*, *SPINT2*, *COL1A1*, *COL1A2*, *COL3A1*, *COL4A1*, *COL4A4*, *COL12A1*, *COL15A1* and *COL16A1*, showed a gradual up‐regulation during the progression of MASLD (Figure 2A, Figure S3).

In contrast, cluster 14, which displayed a reverse correlation with NAS and fibrosis scores, was significantly enriched in metabolic processes, indicating an association between MASLD progression and attenuated liver metabolism. The down‐regulated genes in this cluster were particularly enriched in processes such as organic acid catabolic process (*p*.adjust = 2.57E‐22), carboxylic acid catabolic process (*p*.adjust = 2.57E‐22), small molecule catabolic process (*p*.adjust = 5.28E‐22), alpha‐amino acid metabolic process (*p*.adjust = 6.03E‐20), fatty acid metabolic process (*p*.adjust = 2.60E‐10) and alcohol metabolic process (*p*.adjust = 1.35E‐09). Notably, genes encoding enzymes of the Cytochrome P450 superfamily, including *CYP1A2*, *CYP2C19*, *CYP2J2*, *CYP2E1*, *CYP4A11*, *CYP4A22*, *CYP4F11*, *CYP2C8* and *CYP3A4*, were down‐regulated with increasing MASLD severity (Figure 2B, Figure S4). For a comprehensive list of all enriched GO terms for genes in cluster 4 and 14, please refer to Table S2.

In addition, we developed a MASLD gene expression database (MASLD‐DB) to facilitate the exploration and comparison of all identified protein‐coding genes based on MASLD severity. The MASLD‐DB (https://dreamapp.biomed.au.dk/NAFLD/) was constructed using the ShinyCell framework [40], which was specifically designed for convenient exploration and sharing of single‐cell transcriptome data.

### 3.4 Identification of Candidate Diagnostic Biomarkers

We employed an additional complementary strategy to further refine our list of candidate genes. In this approach, we first analysed the plasma protein levels from a MASLD cohort in a proteomics dataset (PXD011839) [29]. We selected proteins that showed positive correlations with increasing MASLD severity (avg_log2FC > 0) and proteins that showed negative correlations. As a result, we identified 148 up‐regulated proteins and 114 down‐regulated proteins associated with increasing MASLD severity (Figure 2C,D).

The secretome, which consists of secreted proteins, has emerged as a valuable resource for disease diagnostics [41, 42, 43]. In our study, we aim to identify potential diagnostic markers among the candidate genes, by comparing our gene clusters with the secretome database from the Human Protein Atlas [44]. This cross‐analysis revealed a total of 349 genes encoding secreted proteins, with 249 genes showing up‐regulation and 100 genes showing down‐regulation (Figure 2E). Notably, our approach successfully identified a comprehensive list of previously known MASLD diagnostic and prognostic markers, including *ADAMTSL2* [45], *AEBP1* [46] and *BGN* [47] (Table S3), further validating the effectiveness of our approach.

Next, we intersected the protein‐encoding genes of these proteins with the secretome genes and the candidate genes generated from our RNA‐seq analysis. Through this cross‐comparison, we identified 16 up‐regulated secreting genes (*A2M*, *C7*, *COL6A3*, *COLEC11*, *ENPP2*, *FBLN1*, *FBN1*, *FCGBP*, *IGFBP6*, *LCN2*, *LUM*, *MMP2*, *PAPLN*, *PTGDS*, *QSOX1*, *VWF*) and 22 down‐regulated secreting genes (*AZGP1*, *C1RL*, *C4BPA*, *C6, C8B*, *CFHR3*, *CNDP1*, *F2*, *GC*, *HP*, *HPR*, *IL1RAP*, *ITIH1*, *ITIH2*, *ITIH4*, *KLKB1*, *PON3*, *SERPINA10*, *SERPINC1*, *SERPING1*, *SMPDL3A*, *TTR*) associated with increasing MASLD severity in both the RNA‐seq and proteomics data (Figure S5).

### 3.5 QSOX1 and IL1RAP Are Promising Biomarkers for MASLD Severity

**FIGURE 3:**
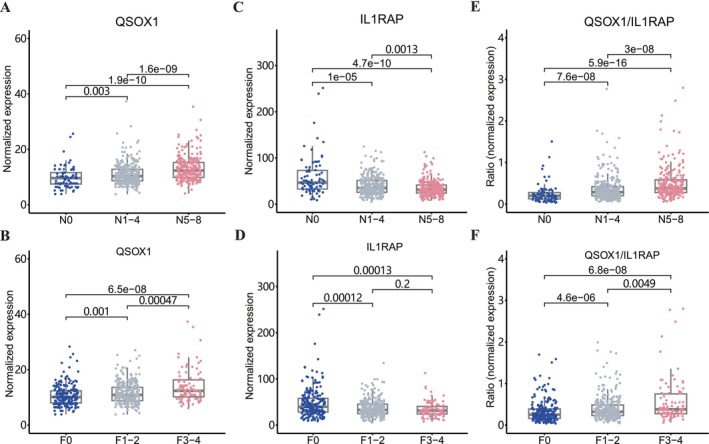
Relationship between QSOX1/IL1RAP and NAS/fibrosis scores in integrative RNA‐seq data of the human liver. (A) Box plot of QSOX1 gene expressions grouped by NAS scores (N0, N1‐4, N5‐8). (B) Box plot of QSOX1 gene expressions grouped by fibrosis stages (F0, F1‐2, F3‐4). (C) Box plot of IL1RAP gene expressions grouped by NAS scores (N0, N1‐4, N5‐8). (D) Box plot of IL1RAP gene expressions grouped by fibrosis stages (F0, F1‐2, F3‐4). (E) Box plot of QSOX1/IL1RAP gene expression ratio grouped by NAS scores (N0, N1‐4, N5‐8). (F) Box plot of QSOX1/IL1RAP gene expression ratio grouped by fibrosis stages (F0, F1‐2, F3‐4). Statistical testing was performed using the Wilcoxon rank sum test, with *p*‐values shown in the plot.

To demonstrate the applicability of our MASLD‐DB and validate the association of differential gene expression with increasing MASLD severity, we selected two representative genes, *QSOX1* and *IL1RAP*, which showed positive and negative correlations with increasing MASLD severity, and their roles as biomarkers were further explored and compared to other MASLD biomarkers (Figure S5). We examined their expression levels in comparison to patients with a NAS or fibrosis score of 0 (N0 or F0). The expression of *QSOX1* was significantly correlated with the severity of MASLD compared to N0 or F0 patients: N1‐4 (*p* = 0.003), N5‐8 (*p* = 1.9E‐10), F1‐2 (*p* = 0.001), F3‐4 (*p* = 6.5E‐8) (Figure 3A,B). On the other hand, *IL1RAP* expression was significantly lower in patients with increased MASLD severity compared to N0 or F0: N1‐4 (*p* = 1E‐5), N5‐8 (*p* = 4.7E‐10), F1‐2 (*p* = 0.00012), F3‐4 (*p* = 0.00013) (Figure 3C,D).

Since *QSOX1* and *IL1RAP* exhibited opposite correlations with MASLD severity, we further explored whether the ratio of QSOX1/IL1RAP could better distinguish between patient groups. Our results showed that compared to N0 or F0 patients, the ratio of QSOX1 to IL1RAP mRNA levels showed even greater separation: N1‐4 (*p* = 7.6E‐8), N5‐8 (5.9E‐16), F1‐2 (4.6E‐6), F3‐4 (6.8E‐8) (Figure 3E,F). These findings suggest that the QSOX1/IL1RAP ratio has the potential as a biomarker for diagnosing MASLD severity.

### 3.6 Validation of Plasma QSOX1/IL1RAP Levels as Biomarkers for MASLD Severity With MASLD Proteomics Cohort

**FIGURE 4:**
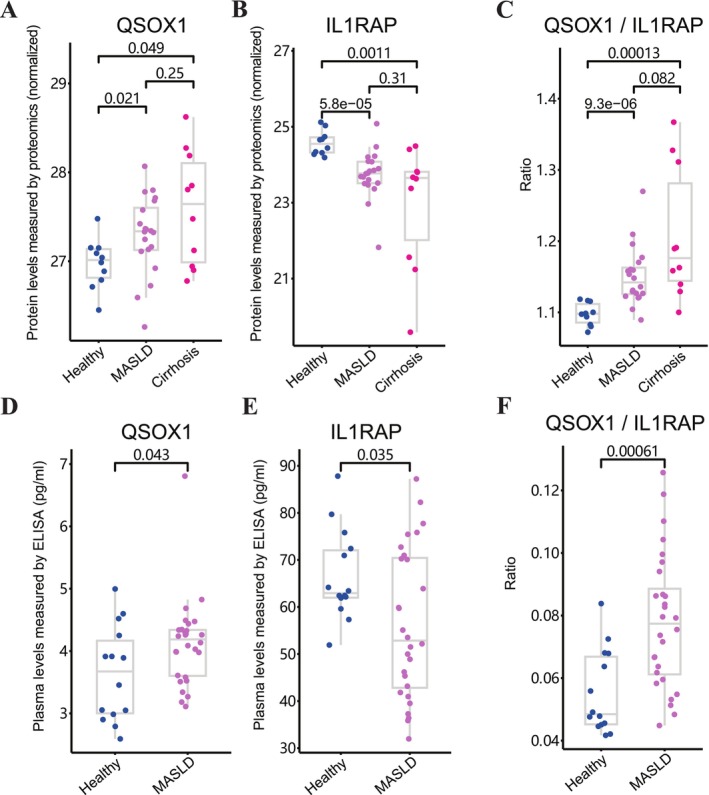
Comparison of plasma protein QSOX1/IL1RAP between healthy individuals and MASLD at various stages. (A, B) Box plots of plasma QSOX1 (A) and IL1RAP (B) protein levels in healthy individuals, MASLD patients, and cirrhosis patients quantified by mass spectrometry in the MASLD proteomics cohort. (C) Box plots of plasma QSOX1 and IL1RAP protein ratios in healthy controls, MASLD patients, and cirrhosis patients quantified by mass spectrometry in the MASLD proteomics cohort. (D, E) Box plots of plasma QSOX1 (D) and IL1RAP (E) protein levels in healthy controls and MASLD groups (pg/mL) measured by ELISA. (F) Box plot of plasma QSOX1 and IL1RAP protein ratio. Statistical testing was performed using the Wilcoxon rank sum test, with *p*‐values shown in the plot.

To further validate the potential of QSOX1 and IL1RAP as biomarkers for MASLD severity, we analysed the plasma levels of QSOX1 and IL1RAP in a MASLD proteomics cohort (PXD011839) previously conducted by Niu L and colleagues [29]. Consistent with our liver RNA profiling results in livers, the analysis of plasma proteomics data from this independent MASLD cohort showed a significant increase in plasma QSOX1 levels in patients with MASLD (Wilcoxon rank sum test, *p* = 0.021) and cirrhosis (*p* = 0.049) compared to healthy controls (Figure 4A). Conversely, IL1RAP levels were significantly reduced in patients with MASLD (*p* = 5.8E‐5) and cirrhosis (*p* = 0.0011) (Figure 4B). Moreover, when considering the combined marker of plasma QSOX1/IL1RAP ratio, it demonstrated even greater significance in distinguishing MASLD (*p* = 9.3E‐6) and cirrhosis (*p* = 0.00013) patients from the control group, compared to using QSOX1 or IL1RAP alone (Figure 4C).

To access the diagnostic sensitivity and specificity of QSOX1, IL1RAP and their ratio for MASLD severity, we conducted ROC curve analysis using the ‘MedCalc’ tool [30]. The sample sizes for each comparison were evaluated and are listed in Table S4. The AUROC of the QSOX1/IL1RAP ratio for distinguishing MASLD patients from healthy controls was 0.95, with a cutoff value of 1.12. The sensitivity was determined to be 90%, and the specificity was 100%. Notably, the efficacy of the QSOX1/IL1RAP ratio was superior to that of IL1RAP alone (AUROC = 0.92) or QSOX1 alone (not significant). Similarly, when assessing the differentiation between cirrhosis patients and healthy controls, the AUROC of the QSOX1/IL1RAP ratio was 0.96, with a cutoff value of 1.12. The sensitivity was 90%, and the specificity was 100%.

These results indicate that the QSOX1/IL1RAP ratio holds promise as a highly effective biomarker for diagnosing MASLD severity, surpassing the individual biomarkers alone and maintaining better sensitivity and specificity in distinguishing MASLD patients and cirrhosis patients from healthy individuals.

### 3.7 Validation of QSOX1 and IL1RAP as Biomarkers for MASLD in Another Patient Cohort

To further validate the utility of QSOX1 and IL1RAP as biomarkers for MASLD, we conducted a validation study in healthy controls and MASLD patients recruited from the Department of Liver Disease of Shenzhen Traditional Chinese Medicine Hospital. Plasma samples were collected from 14 healthy subjects and 28 newly diagnosed MASLD patients. Clinical and biochemical information for the healthy controls and MASLD patients can be found in Tables S5 and S6.

We measured plasma levels of QSOX1 and IL1RAP using an enzyme‐linked immunosorbent assay (ELISA). Consistent with our previous findings, plasma levels of QSOX1 (Wilcoxon rank sum test, *p* = 0.043), IL1RAP (*p* = 0.035) and the QSOX1/IL1RAP ratio (*p* = 0.00061) were significantly different between MASLD patients and controls (Figure 4D–F).

To assess the diagnostic value of QSOX1 and IL1RAP as non‐invasive biomarkers for MASLD by ELISA, we calculated the AUROC of the QSOX1/IL1RAP ratio in the ELISA test to distinguish MASLD patients from healthy controls. The QSOX1/IL1RAP ratio exhibited an AUROC of 0.82. Using a cutoff of 0.05, the sensitivity was 93% and the specificity was 57%. In comparison, the AUROC of the QSOX1/IL1RAP ratio quantified by ELISA showed less efficacy in distinguishing MASLD patients from healthy controls (Table S4), which may be attributed to the sensitivity of protein quantification methods and small sample size.

**FIGURE 5:**
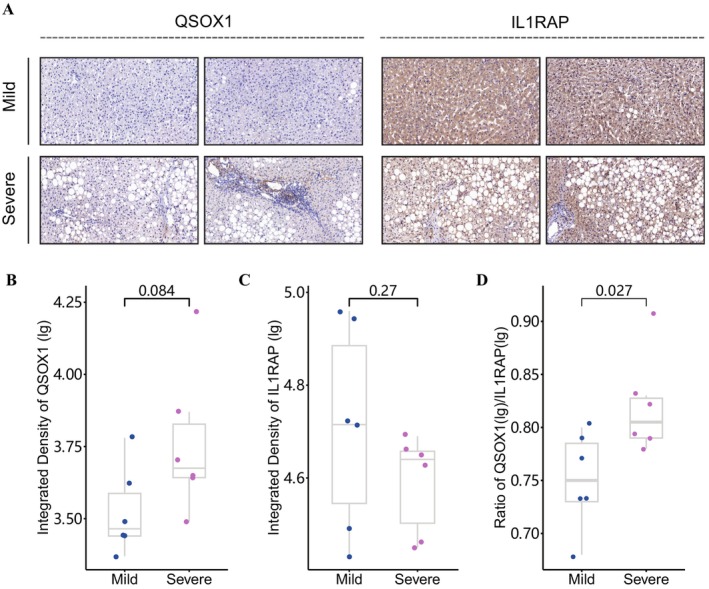
Quantification of liver QSOX1 and IL1RAP levels in MASLD patients by IHC. (A) Representative IHC images of QSOX1 and IL1RAP in liver biopsies from mild MASLD patients (N0‐4, F0‐2) and severe MASLD patients (N5‐8, F3‐4). (B) The integrated density of QSOX1 IHC. Box plots showing the log10 value. (C) The integrated density of IL1RAP IHC. Box plots showing the log10 value. (D) Box plot of QSOX1 and IL1RAP ratio of the integrated density quantified with IHC. Statistical testing was performed using *t*‐test, with *p*‐values shown in the plot.

To further validate the association between QSOX1 and IL1RAP protein levels and MASLD severity, we assessed their levels in liver biopsies from mild and severe MASLD patients using IHC. Our results consistently demonstrated a significant correlation between QSOX1 and IL1RAP levels and MASLD severity (Figure 5A). Quantification of QSOX1 and IL1RAP levels based on IHC confirmed that the QSOX1/IL1RAP ratio (*p* = 0.027) could distinguish the severe MASLD group (*n* = 6; NAS 5–8, fibrosis score 3–4) from the mild MASLD group (*n* = 6; NAS 0–4, fibrosis score 0–2) (Figure 5B,C). Collectively, these findings support the above data that the QSOX1/IL1RAP ratio holds promise as a biomarker for the early diagnosis and prediction of MASLD severity.

## 4 Discussion

This study is the first to integrate publicly available RNA‐seq datasets from over 600 patients with MASLD with varying stages of disease severity, combined with proteomics data analysis of publicly available datasets. The key findings suggest that the QSOX1 and IL1RAP, and particularly the QSOX1/IL1RAP ratio hold promise as potential biomarkers for MASLD severity assessment. These results align with recent research highlighting the importance of different transcriptional profiles specific to NAS and fibrosis scores, offering valuable insights into the molecular mechanisms driving disease progression from simple steatosis to inflammation and fibrosis [21, 24].

### 4.1 The Advantages of Utilizing Integrated RNA‐Seq Data for Investigating MASLD Biomarkers

Despite the growing availability of RNA‐seq data in this field, many original studies have been limited by small sample sizes and biased sample distribution, making it challenging to accurately decipher transcriptional differences across various stages of MASLD [18, 20, 21, 22, 24]. Several studies have attempted to identify diagnostic biomarkers and potential drug targets. For instance, Brosch et al. conducted a positional analysis of transcriptomes across three micro‐dissected liver zones from 19 MASLD patients [18]. Suppli et al. demonstrated that immunohistochemical markers offer greater objectivity in distinguishing hepatocyte injury between NASH and NAFL [20]. In the pursuit of diagnostic genes and novel drug targets, Hoang et al. studied 6 histologically normal and 72 MASLD patients, while Pantano et al. studied 31 histologically normal and 112 MASLD patients. These studies revealed that specific cell proportions and candidate gene signatures can accurately predict fibrosis stage and disease progression [21, 24]. Likewise, Govaere et al. observed the correlation between gene expression and histology in a cohort of 10 controls and 206 MASLD patients [22]. In contrast to the studies above that identified sets of potential biomarker genes, Kozumi et al. validated thrombospondin 2 (THBS2) as a noninvasive biomarker for MASLD. They confirmed its potential in identifying the disease stages among 98 MASLD patients, and the serum levels of its encoded protein TSP‐2, measured by ELISA, showed an AUROC of 0.78 in the diagnosis of NASH among 213 patients with biopsy‐proven MASLD [25]. The major challenge of combining and analysing these diverse datasets lies in achieving homogeneous processing, which requires substantial time and computational resources [48, 49]. To generate more robust and compelling results, we employed unbiased integration of comprehensive MASLD data to profile the liver transcriptome across a broad spectrum of MASLD severity in our study, incorporating all the aforementioned samples.

### 4.2 The Superiority of QSOX1 and IL1RAP as Potential Biomarkers of MASLD

The high prevalence and associated risks of MASLD have driven global efforts to identify improved diagnostic biomarkers. However, most existing biomarkers are primarily suited for evaluating fibrosis [3, 50, 51, 52]. The Fibrosis‐4 (FIB‐4) test commonly used in clinical practice is sub‐optimal for screening purposes, as it carries the risks of both overdiagnosis and false negatives, particularly in patients at risk of chronic liver disease [8]. Although the patented ELF test was highly recommended for ruling out advanced fibrosis, it comes with higher costs. In a recent meta‐analysis, FIB‐4 showed AUROC of 0.80 (95% CI 0.76–0.83) for significant fibrosis (≥ F2) and 0.84 (95% CI: 0.81–0.87) for advanced fibrosis (≥ F3) [53]. The ELF test performed slightly better with AUC of 0.81 (95% CI 0.66–0.89) for significant fibrosis (≥ F2) and 0.83 (95% CI 0.71–0.90) for advanced fibrosis [54].

Several steatosis scores, such as the SteatoTest and the fatty liver index (FLI), have been proposed for steatosis detection, but they do not provide substantial additional information beyond routine clinical, laboratory and imaging examinations conducted in patients suspected of having MASLD [8]. Non‐coding RNAs (ncRNAs), which exhibit aberrant expression associated with MASLD, have emerged as potential biomarkers for MASLD pathology and circulating ncRNAs including miR‐122 and lncRNAs are proposed as potential biomarkers for MASLD severity and progression [55, 56, 57, 58, 59, 60, 61]. Despite the development of new biomarkers, there is still uncertainty surrounding their predictive value, underscoring the urgent need to develop novel, cost‐effective and efficient biomarkers with high sensitivity and specificity for MASLD prediction and monitoring [4, 62].

The approach by Hoang et al. [21] centered on identifying genes with diverse expressions associated with MASLD severity, inspired us to develop our gene clustering method. Our approach surpasses the constraints of conventional RNA‐seq data analysis, which predominantly relies on pairwise comparisons. Instead, it classifies genes according to their dynamic expression patterns, enabling a more comprehensive and dynamic perspective of molecular alterations as MASLD progresses. This method has the potential to map MASLD severity and progression solely through gene expressions, thus avoiding invasive procedures like liver biopsies. Moreover, the gene‐based scoring system can forecast MASLD progression, facilitating early interventions for patients at risk of advancing to severe disease stages.

QSOX1 has been proposed as a diagnostic biomarker for MASLD due to its role in lipid metabolism and expression in various tissues, particularly in quiescent fibroblasts [18, 63, 64]. IL1RAP, localized to vesicles and cytosol and secreted into the bloodstream, shows liver‐specific RNA expression in hepatocytes [44]. Based on their secretome profiles, QSOX1 and IL1RAP were selected as a potential biomarker combination. While prior studies have examined the involvement of these proteins in MASLD [16, 65], no GWAS or proteomic data currently link genetic variants in *QSOX1* or *IL1RAP* to protein levels or MASLD phenotypes. However, *IL2RAP*, a related gene, harbours SNPs associated with obesity and lipid deposition traits in large‐scale GWAS, including studies showing its plasma levels are influenced by polygenic scores for BMI [66]. For *QSOX1*, although no MASLD‐specific genetic associations have been reported, functional proteomic data demonstrate a strong correlation between plasma QSOX1 levels and fibrosis stage in alcohol‐related liver disease (Spearman *r* = 0.6) [67]. This supports our transcriptomic findings and suggests a broader role for QSOX1 in liver fibrogenesis beyond alcohol‐related disease.

The potential of QSOX1, IL1RAP and their ratio as biomarkers for MASLD was demonstrated through the analysis of public RNA‐seq and proteomics data, ELISA tests conducted on patients' plasma and IHC performed on fixed liver slides. We are well aware that the ratio reflecting the detected up‐ and down‐regulation of genes improves the diagnostic accuracy without having a direct pathogenic implication. However, our findings suggest that QSOX1, IL1RAP and their ratio with higher AUROC values for MASLD diagnosis hold promise as effective biomarkers for MASLD.

### 4.3 Limitation and Future Prospects

The current study possesses several strengths, including the integration and processing of RNA‐seq data from over 600 MASLD patients with varying degrees of MASLD severity, as well as validation using proteomics data and samples from MASLD patients and controls. Furthermore, the well‐established database with a user‐friendly interface could benefit the research community in exploring differentially expressed genes in MASLD at various stages. However, there are also limitations to consider. For instance, some samples in the GSE126848 and GSE167523 datasets lacked individual NAS and fibrosis scores. To address this issue, we standardized scores based on their categories in the original articles, and the impact on the results was deemed negligible due to the provision of general stages and unsupervised gene clustering. Further, sensitivity analysis examining the raw QSOX1/IL1RAP ratio across assigned NAS and fibrosis score groups in all samples showed consistent positive associations with both NAS and fibrosis scores. Machine learning, an essential tool for biomarker validation and sample classification validation, should be employed to train large cohorts of biopsy‐proven patients with MASLD and healthy controls. However, this would require an extended recruiting period [68] to determine the sensitivity and specificity of the QSOX1/IL1RAP ratio for MASLD diagnosis and staging. It is necessary to explicitly state that the biomarker ratio does not demonstrate a mechanistic causal relationship, but rather is intended as a diagnostic indicator.

Although newer technologies such as single‐cell RNA sequencing (scRNA‐seq) and spatial sequencing have gained popularity, RNA‐seq still serves as a valuable tool in uncovering the pathogenesis of MASLD [17]. Computational analysis limitations make it impractical for large cohort research, and single‐cell suspension processing may affect cell abundance and cell type representation, particularly in hepatic ballooning cells in MASLD [69]. Single‐nuclei RNA sequencing (snRNA‐seq) captures cell frequency more accurately than scRNA‐seq but captures lower gene expression. Spatial transcriptomics and proteomics have limitations for discovering invasive biomarkers of MASLD as they focus on small sampling areas [15]. The combination of all these biological tools holds potential for future research.

In conclusion, through a novel approach of unsupervised gene clustering performed on integrated RNA‐seq data, we have discovered a significant association between QSOX1 and IL1RAP levels and MASLD severity, with their ratio showing potential as a non‐invasive biomarker for diagnosing and assessing the severity of MASLD. Validation of our plasma‐level findings in larger cohorts of liver biopsies is required, but it holds promise as a new tool to diagnose MASLD severity and reduce the need for liver biopsies. Our approach may lead to the discovery of more MASLD biomarkers, and the ratios of other up‐regulated and down‐regulated genes associated with increasing MASLD severity also have the potential to be verified as potential biomarkers.

## Author Contributions

Yonglun Luo, Henning Grønbæk, Wenfeng Ma and Lin Lin designed the study and interpreted the data. The analysis strategy has been developed by Lin Lin and Wenfeng Ma. Wenfeng Ma, Jinrong Huang, Benqiang Cai, Minling Lv, Xin Zhong, Shaomin Xu and Mikkel Breinholt Kjær collected and assembled the data. Wenfeng Ma drafted the manuscript. Wenfeng Ma, Jinrong Huang and Lei Cheng performed data analysis and/or interpretation. Technical support: Yonglun Luo, Lin Lin, Jinrong Huang, Xiaozhou Zhou, Mumin Shao and Xuewen Yu. Study participant inclusion: Xiaozhou Zhou, Wenfeng Ma, Benqiang Cai, Minling Lv, Xin Zhong, Shaomin Xu, Bolin Zhan, Qun Li, Qi Huang, Mengqing Ma. All authors reviewed and approved the final version of the manuscript.

## Funding

This research was funded by the Shenzhen Science and Technology Project and Sanming Project of Medicine in Shenzhen, China (Grant SZSM201612074, JCYJ20210324120405015).

## Conflicts of Interest

Henning Grønbæk has received research grants from Abbvie, Intercept, ARLA Food for Health, ADS AIPHIA Development Services AG. Consulting Fees from Ipsen, NOVO, Pfizer. Lecturer for AstraZeneca and EISAI; and on Data Monitoring Committee at CAMURUS AB. All other authors have no conflicts of interest to declare.

## Supporting information


**Figure S1:** t‐SNE visualization of gene clusters.
**Figure S2:** Heatmap presentation of gene expression profile along MASLD progression.
**Figure S3:** Heatmap presentation of ECM gene expression profile.
**Figure S4:** Heatmap presentation of Cytochrome P450 superfamily gene expression profile.
**Figure S5:** Heatmap presentation of expression profile for 32 biomarker genes.

**Table S1:** RNA sequencing data list (Genome‐wide RNA‐seq data of human MASLD and associated healthy controls collected from the NCBI GEO).

**Table S2:** List of secreting protein‐encoding genes in cluster 4, and 14 of RNA‐seq analysis.

**Table S3:** Genes encoding secreted proteins, with 249 genes showing up‐regulation and 100 genes showing down‐regulation.

**Table S4:** Performance Characteristics of QSOX1, IL1RAP and the QSOX1/IL1RAP ratio in proteomics data and the results of ELISA.

**Table S5:** Metadata for MASLD patients and control participants involved in the ELISA validation study.

**Table S6:** Metadata of detailed information for MASLD patients and control participants involved in the ELISA and IHC validation study.

## Data Availability

The data that support the findings of this study are available from the corresponding author upon reasonable request.
