# Supplementary material for: Gene‐Based Clustering Identifies QSOX1 and IL1RAP as Biomarkers of Metabolic Dysfunction‐Associated Steatotic Liver Disease
Source: Liver Int. 2026 Jul 29;46(9):e70823. doi: 10.1111/liv.70823 (PMC13417051; doi:10.1111/liv.70823)
Supplement: Supplementary file 1 — Figure S1: t‐SNE visualization of gene clusters. Figure S2: Heatmap presentation of gene expression profile along MASLD progression. Figure S3: Heatmap presentation of ECM gene expression profile. Figure S4: Heatmap presentation of Cytochrome P450 superfamily gene expression profile. Figure S5: Heatmap presentation of expression profile for 32 biomarker genes. [file LIV-46-0-s004.docx]

**Supplementary File**

**Gene-based clustering identifies QSOX1 and IL1RAP as biomarkers of metabolic dysfunction-associated steatotic liver disease**

Wenfeng Ma ^1,2,3,4^, Jinrong Huang ^2^, Benqiang Cai ^1,4^, Mumin Shao ^6,7^, Xuewen Yu ^6,7^, Mikkel Breinholt Kjær ^5,8^, Minling Lv ^1,4^, Xin Zhong ^1,4^, Shaomin Xu ^1,4^, Bolin Zhan ^1,4^, Qun Li ^1,4^, Qi Huang ^1,4^, Mengqing Ma ^1,4^, Lei Cheng ^2^, Yonglun Luo ^2,3^*, Henning Grønbæk ^5^*, Xiaozhou Zhou ^1,4^, Lin Lin ^2,3^*

1 Department of Liver Disease, Shenzhen Traditional Chinese Medicine Hospital, Shenzhen, Guangdong 518033, China.

2 Department of Biomedicine, Aarhus University, Aarhus, Denmark.

3 Steno Diabetes Center Aarhus, Aarhus University Hospital, Aarhus, Denmark.

4 Department of Liver Disease, The Fourth Clinical Medical College of Guangzhou University of Chinese Medicine, Shenzhen, 518033, China.

5 Department of Hepatology and Gastroenterology, Aarhus University Hospital, Aarhus, Denmark.

6 Department of Pathology, Shenzhen Traditional Chinese Medicine Hospital, Shenzhen, Guangdong 518033, China.

7 Department of Pathology, The Fourth Clinical Medical College of Guangzhou University of Chinese Medicine, Shenzhen, 518033, China.

8 Department of Clinical Medicine, Aarhus University, Aarhus, Denmark.

* = corresponding author

**This supplementary file contains: Supplementary Figure S1-S5**

**Supplementary Figure S1**


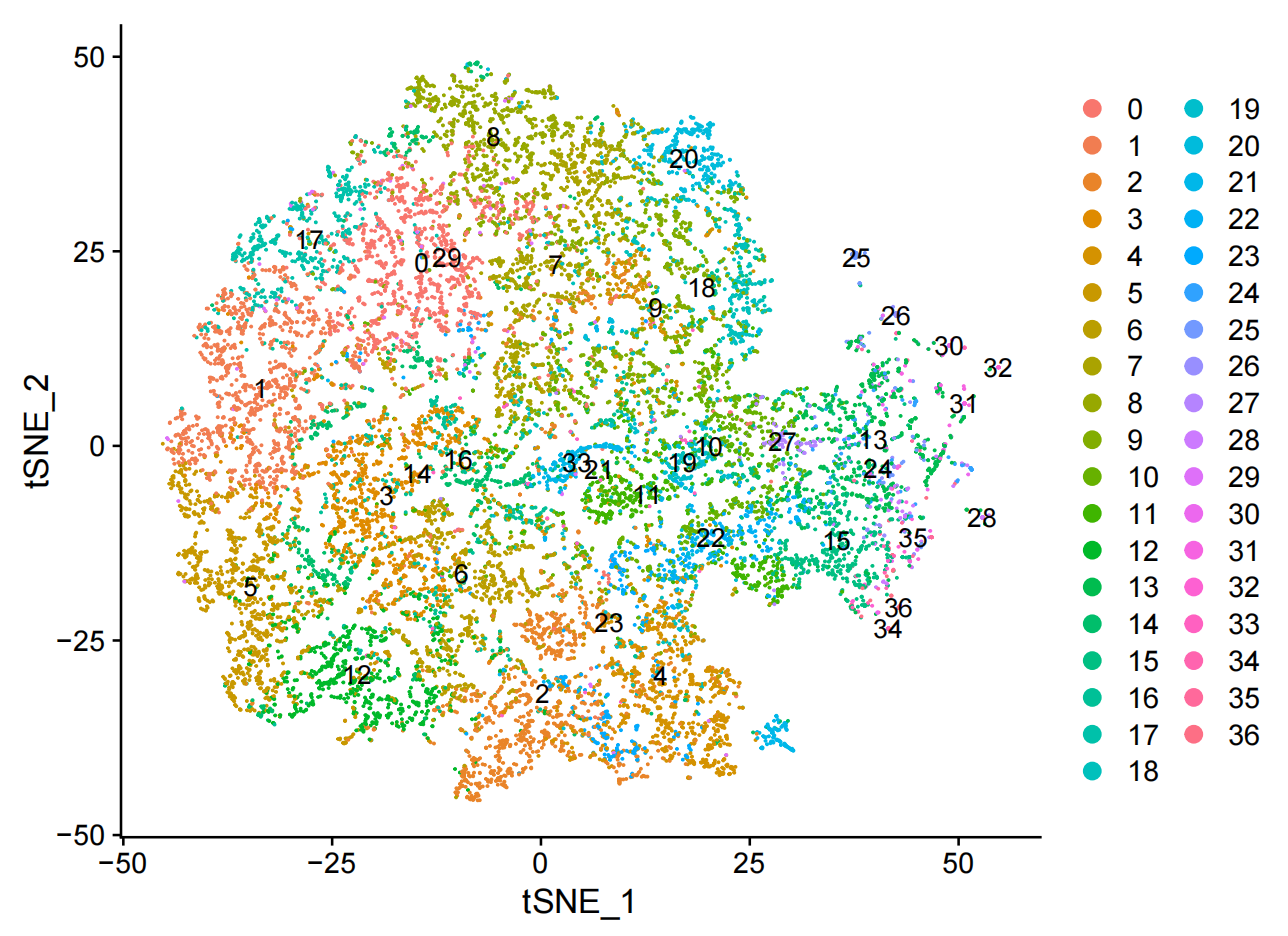


**Supplementary Figure S1. t-SNE visualization of gene clusters**

Visualization of gene expression profiling clusters across MASLD progression with the t-distributed stochastic neighbor embedding (t-SNE) statistical method. Each dot represents one protein coding gene (n = 17,946). A graph-based clustering approach was used. The dimensions of reduction were set to 1:20 and visualized with a resolution of 2.3.

**Supplementary Figure S2**


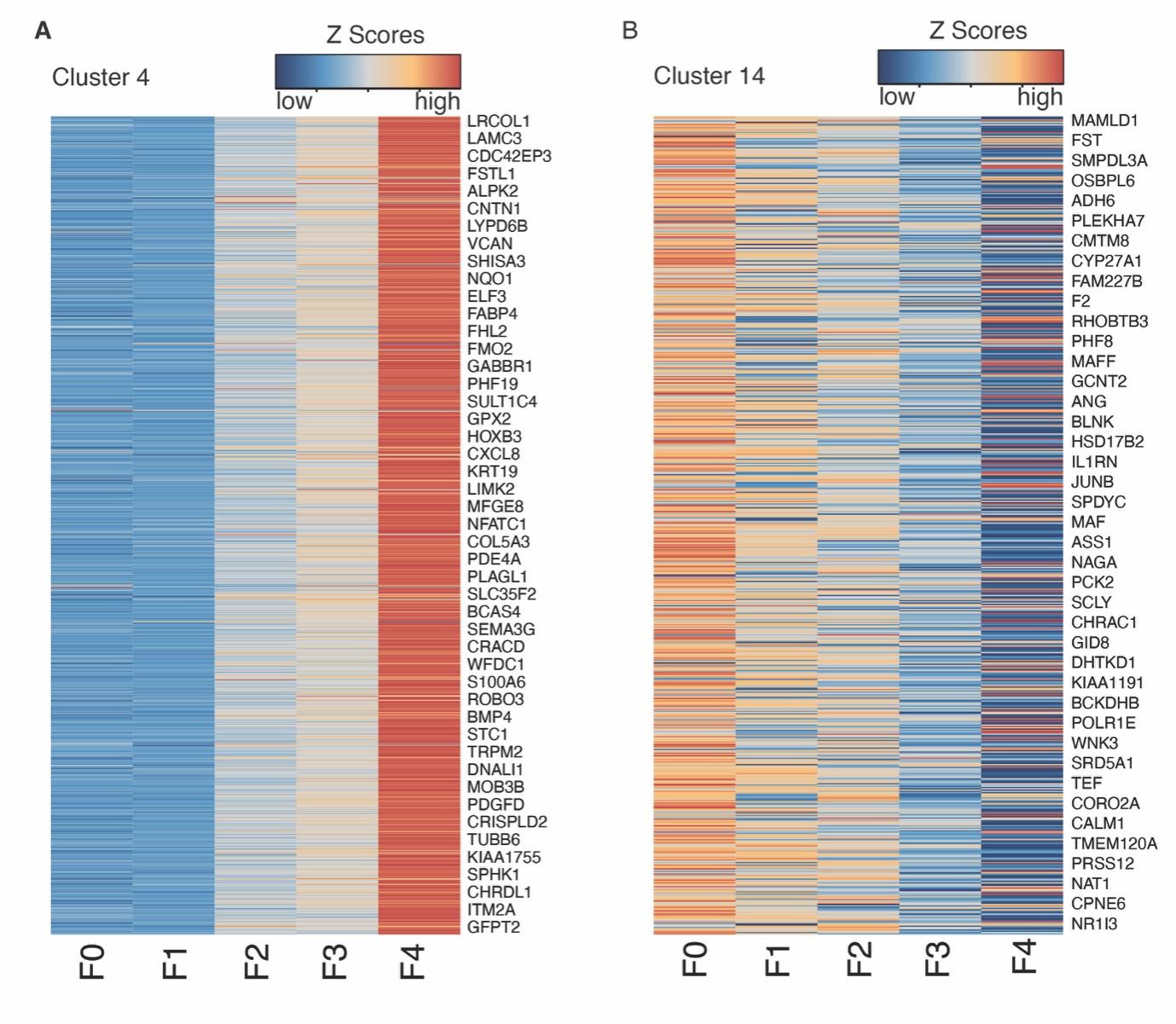


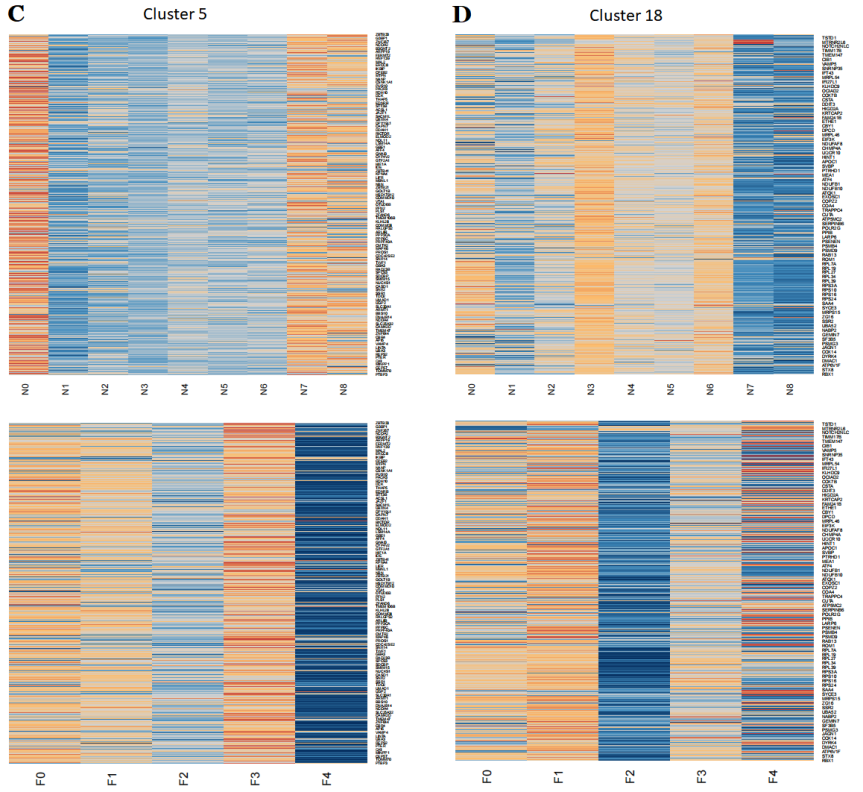


**Supplementary Figure S2 Heatmap presentation of gene expression profile along MASLD progression.**

A. Heatmap presentation of 1021 up-regulated genes in cluster 4 associated with increasing fibrosis scores.

B. Heatmap presentation of 643 down-regulated genes in cluster 14 associated with increasing fibrosis scores.

C and D. Contrary to genes in cluster 4 and 14, C and D displayed gene clusters with chaotic gene expression patterns associated with both NAS scores and fibrosis scores.

**Supplementary Figure S3**


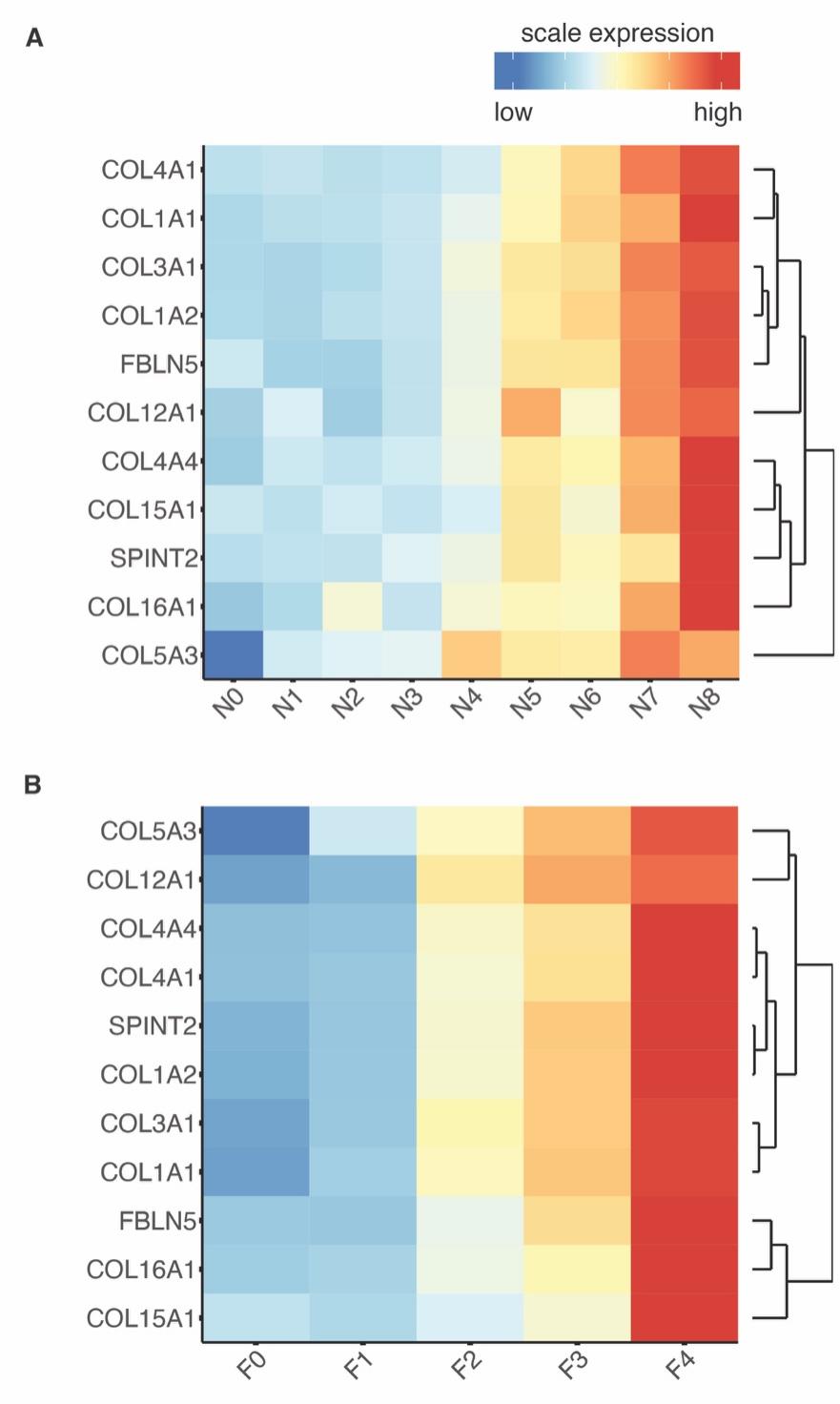


**Supplementary Figure S3 Heatmap presentation of ECM gene expression profile**

A. Scale gene expression profile of multiple genes involved in the ECM process according to NAS scores. B. Scale gene expression profile of multiple genes involved in the ECM process according to fibrosis scores. Genes were clustered based on profile similarity. Genes expression level was scaled for heatmap presentation, also see the MASLD gene expression database (MASLD-DB).

**Supplementary Figure S4**


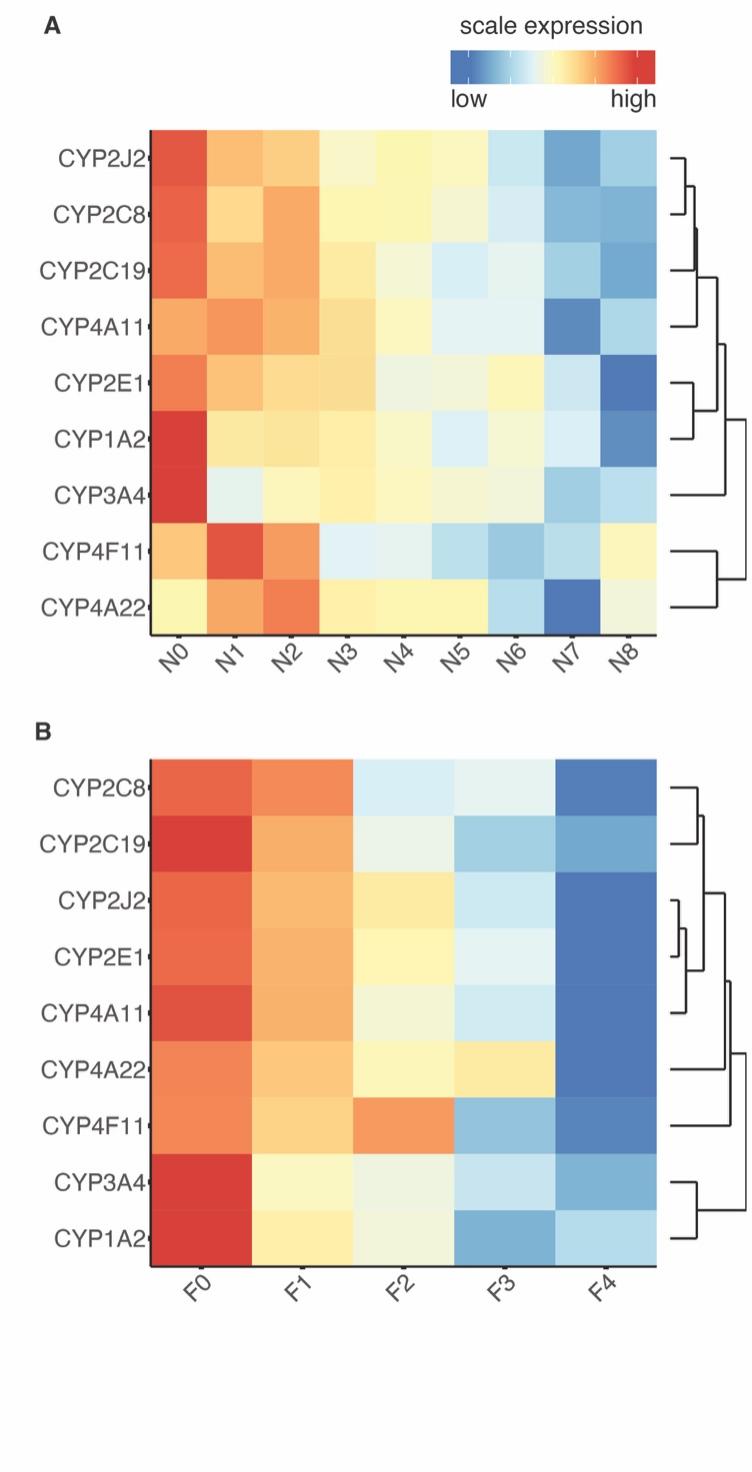


**Supplementary Figure S4 Heatmap presentation of Cytochrome P450 superfamily gene expression profile**

A. Scale gene expression profile of multiple genes involved in the Cytochrome P450 superfamily according to NAS scores. B. Scale gene expression profile of multiple genes involved in the Cytochrome P450 superfamily according to fibrosis scores. Genes were clustered based on profile similarity. Genes expression level was scaled for heatmap presentation (also see the MASLD-DB).

**Supplementary Figure S5**


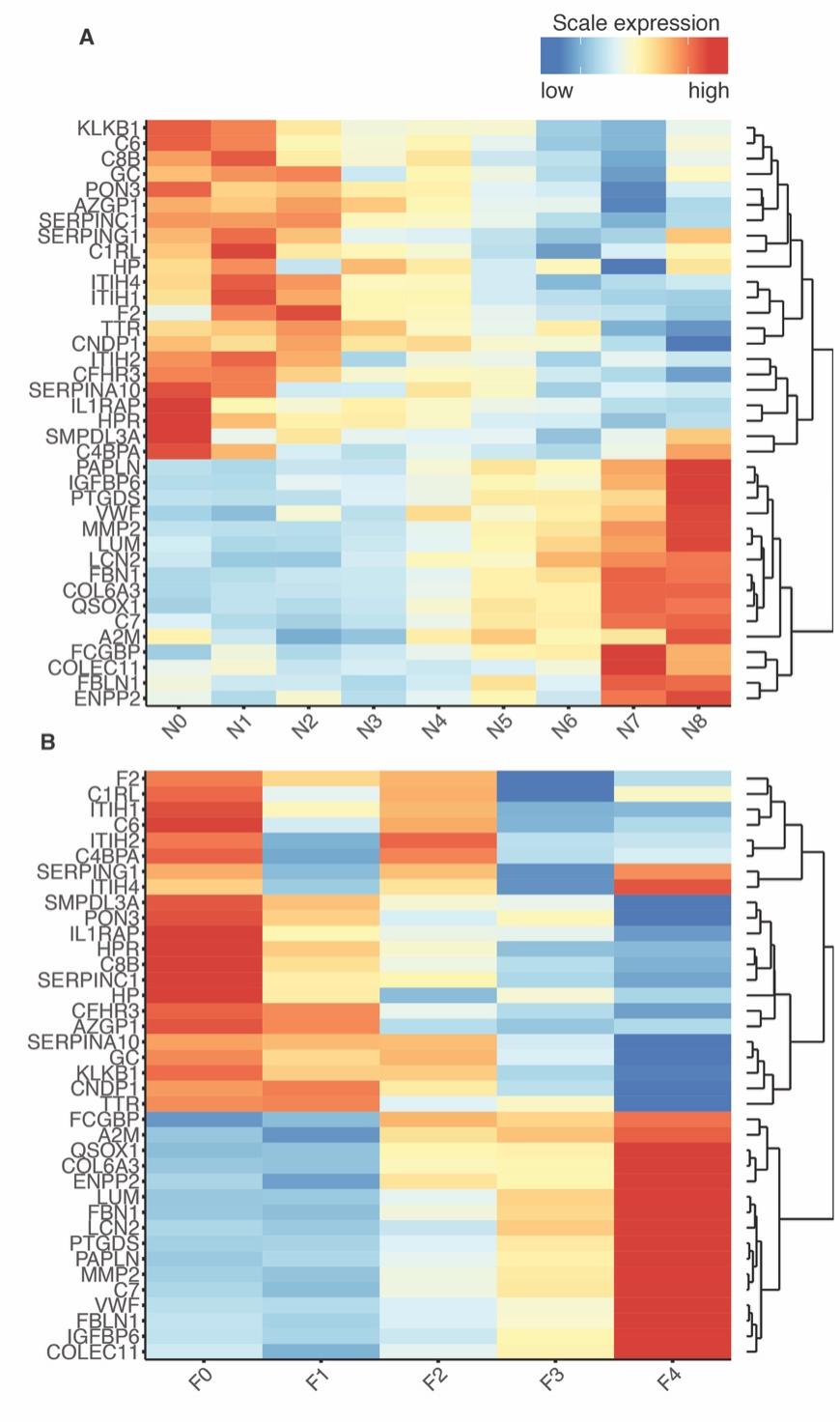


**Supplementary Figure S5 Heatmap presentation of expression profile for 32 biomarker genes**

1. Scale gene expression profile of 38 biomarker genes (16 up-regulation, 22 down-regulation) according to NAS scores. B. Scale gene expression profile of 38 biomarker genes according to fibrosis scores. Genes were clustered based on profile similarity. Genes expression level was scaled for heatmap presentation (also see the MASLD-DB). This figure is related to Figure 2E.

**The caption of the supplementary table 1-6**

**Supplementary Table 1**

RNA sequencing data list (Genome-wide RNA-seq data of human MASLD and associated healthy controls collected from the NCBI GEO)

**Supplementary Table 2**

List of secreting protein-encoding genes in cluster 4, and 14 of RNA-seq analysis.

**Supplementary Table 3**

Genes encoding secreted proteins, with 249 genes showing up-regulation and 100 genes showing down-regulation

**Supplementary Table 4**

Performance Characteristics of QSOX1, IL1RAP, and the QSOX1/IL1RAP ratio in proteomics data and the results of ELISA

**Supplementary Table 5**

Metadata for MASLD patients and control participants involved in the ELISA validation study.

**Supplementary Table 6**

Metadata of detailed information for MASLD patients and control participants involved in the ELISA and IHC validation study.
